# 6-(4-Pyridyl)Azulene Derivatives as Hole Transport Materials for Perovskite Solar Cells

**DOI:** 10.3390/ma18071400

**Published:** 2025-03-21

**Authors:** Yuanqing Sun, Zhangyan Wang, Tianyi Geng, Xinyue Liu, Yangyang Su, Yi Tian, Ming Cheng, Hongping Li

**Affiliations:** 1 Institute for Energy Research, Jiangsu University, Zhenjiang 212013, China; sun_yuanqing@foxmail.com (Y.S.); 2212393010@stmail.ujs.edu.cn (X.L.); mingcheng@ujs.edu.cn (M.C.); 2 Institute for Advanced Materials, School of Materials Science and Engineering, Jiangsu University, Zhenjiang 212013, China; 18297498105@163.com; 3 School of Energy and Power Engineering, Jiangsu University, Zhenjiang 212013, China

**Keywords:** hole transport materials, azulene, photovoltaic, perovskite solar cell

## Abstract

Azulene has been attracting much attention as a charge transfer material in organic electronics due to its inherent large dipole moment and small band gap, but its application in perovskite solar cells (PSCs) is very limited. Herein, azulene was applied as the core acceptor for hole transport materials (HTMs), and two molecules named Azu-Py-DF and Azu-Py-OMeTPA were designed and synthesized, in which 4-pyridyl was introduced on the 6-position of the 1,3-substituted azulene core to adjust energy levels. The different spatial orientations of pyridine and the azulene core improve the solubility and reduce the crystallinity of the material, which is conducive to creating a thin film morphology. Azu-Py-OMeTPA exhibited good hole and electron mobility compared with standard Spiro-OMeTAD. Applied as an HTM in PSCs, the Azu-Py-OMeTPA-based device achieved a power conversion efficiency (PCE) of 18.10%, which is higher than that of the 6-position unsubstituted counterpart. Nevertheless, the anticipated passivation effect of the 4-pyridyl group was diminished due to the electron-deficient nature of azulene’s seven-membered ring. These results demonstrate that optimizing the structure of azulene-based HTMs can significantly alter molecular spatial structure, film formation properties, electron delocalization characteristics and charge transport, and can lead to improved device performance, providing insights for the future design of novel HTMs.

## 1. Introduction

Perovskite materials have shown superior performance in photovoltaic technology due to their advantages of having a large absorption coefficient, long diffusion length, wide absorption range, and low manufacturing cost [1,2,3,4,5,6,7], so they are widely regarded as a promising new-generation photovoltaic technology, and their application would serve as a key method for dealing with environmental issues such as climate change [8,9,10] by reducing fossil fuel consumption. At present, the power conversion efficiency (PCE) of single-junction perovskite solar cells exceeds 26% [11], which is approaching the efficiency of silicon solar cells. Typical n-i-p PSC devices usually adopt a layer-by-layer sandwich-like structure, which contains conductive glass, an electron transport layer, a perovskite light absorption layer, a hole transport layer, and a Au/Ag metal anode from the device’s bottom to top. Because photogenerated holes generally move more slowly than electrons, hole transport layers play an important role and have a crucial impact on PCE by accelerating hole extraction and transportation [12,13,14,15,16]. Hole transport layers also block electrons from the metal anode to prevent charge recombination, ensuring the efficient photoelectric conversion of PSCs. To date, the leading hole transport material is an organic compound with a spiro structure, notably 2,2′,7,7′-tetrakis(N,N-p-dimethoxyphenylamino)-9,9′-spirobifluorene (Spiro-OMeTAD). Despite its prominence, the commercial use of Spiro-OMeTAD is hindered by complex synthesis processes and elevated costs. As a result, significant efforts have been devoted to designing alternative HTMs. A common strategy is to modulate the energy level, hole mobility, conductivity, and film morphology to achieve better interfacial contact and hole transport. HTMs with a donor–acceptor–donor (D-A-D) structure have advantages in terms of enhancing PCE due to their properties which can be easily tuned by means of chemical methods [17,18,19,20,21].

Azulene is an aromatic bi-heterocyclic isomer of naphthalene with a chemical structure of C_10_H_8_; it has excellent optoelectronic properties due to its inherent large dipole moment (1.08 D) and small band gap between the ground state and the first excited state, which originate from its unique structure formed by an electron-rich five-membered ring fused with an electron-deficient seven-membered ring [22,23,24,25]. The highest occupied molecular orbital (HOMO) and lowest unoccupied molecular orbital (LUMO) are separately distributed on the odd positions and even positions, respectively (Figure S1). Thus, the odd positions are electron-rich and usually undergo electrophilic reactions. In contrast, the even positions are electron-deficient and favor nucleophilic reactions. Azulene derivatives have long been used in organic electronics such as organic photovoltaic cells, organic field-effect transistors, nonlinear optical materials, and molecular devices in which the molecular polarity and intermolecular π-π interaction are effectively regulated by chemical modifications, and they have a crucial effect on enhancing the performance of their corresponding devices.

Despite its excellent charge transportation property, the application of azulene in PSCs is very limited and has only been reported in a few papers by the research groups of Wakamiya and Kymakis [26,27,28,29]. In these studies, azulene-based HTMs showed quite impressive performance due to the strong intermolecular interaction and high hole transportation induced by the planar molecular structure and large dipole moment of azulene, indicating its great potential as a building block for HTMs. Our group previously synthesized novel D-A-D-type HTMs based on a 1,3-substituted azulene core, in which azulene was applied as the electron acceptor core, and they achieved higher hole mobility and conductivity compared to Spiro-OMeTAD, but device performance was mainly limited by poor film formation and a moderate PCE of only up to 15.41% [30]. In this study, the properties of azulene-based HTMs were optimized by changing the peripheral groups on the 1- and 3-positions of azulene and further introducing a 4-pyridyl group on the 6-position. The two 1,3,6-trisubstituted azulene molecules were named Azu-Py-DF and Azu-Py-OMeTPA. The different spatial orientations of the 4-pyridyl group and azulene core improved the solubility and further regulated the energy levels of the materials, which are conducive to improving the morphology of the film and enabling the extraction of photogenerated holes. Applied in PSCs, Azu-Py-OMeTPA achieved an improved PCE of 18.10%, higher than that of Azu-Py-DF (15.39%). Although the performance of our azulene-based materials is still lower than that of Spiro-OMeTAD and further molecular structure modifications are needed, we hope that this study serves as a reference for the design and synthesis of azulene-based charge transfer materials.

## 2. Experimental Section

### 2.1. Synthesis and Characterization of HTMs

#### 2.1.1. Synthesis of 6-(4-Pyridyl)Azulene (A2)

To a solution of A1 (6-bromoazulene, 1.5 g, 7.24 mmol) in dioxane/water (30/6 mL), 4-pyridineboronic acid (1.34 g,10.87 mmol), K_2_CO_3_ (5.01 g, 36.22 mmol), and Pd(PPh_3_)_4_ (250 mg, 0.22 mmol) were added. The mixture was stirred at 110 °C under a nitrogen atmosphere for 16 h. The reaction was then diluted with water (150 mL), and the solvent was evaporated under vacuum. The resulting suspension was extracted with ethyl acetate (50 mL × 3), and the organic phase was washed with brine (20 mL), dried over Na_2_SO_4_, concentrated under vacuum, and purified with column (20–40% ethyl acetate in petroleum ether) to afford A2 (1.1 g,74%) as a blue solid. ^1^H NMR (400 MHz, Chloroform-*d*) δ 8.71 (d, *J* = 6.2 Hz, 2H), 8.44 (d, *J* = 10.6 Hz, 2H), 7.98 (dd, *J* = 3.8 Hz, 3.8 Hz, 1H), 7.56 (d, *J* = 6.2 Hz, 2H), 7.47 (d, *J* = 3.8 Hz, 2H), and 7.36 (d, *J* = 10.6 Hz, 2H).

#### 2.1.2. Synthesis of 1,3-Dibromo-[6-(4-Pyridyl)]Azulene (A3)

To a solution of A2 (1.1 g, 5.36 mmol) in dichloromethane (20 mL), N-bromosuccinimide (2.1 g, 11.79 mmol) was added, and the mixture was stirred at 25 °C in dark for 2 h. The reaction was quenched with saturated Na_2_S_2_O_3_(50 mL) and extracted with dichloromethane (30 mL × 2). The organic phase was separated, washed with brine (20 mL), dried over Na_2_SO_4_, concentrated under vacuum, and purified with column (40–60% ethyl acetate in petroleum ether) to afford A3 (1.5 g,77%) as a green solid. ^1^H NMR (400 MHz, Chloroform-*d*) δ 8.75 (d, *J* = 5.1 Hz, 2H), 8.36 (d, *J* = 10.8 Hz, 2H), 7.85 (s, 1H), 7.54 (d, *J* = 6.2 Hz, 2H), and 7.43 (d, *J* = 10.8 Hz, 2H).

#### 2.1.3. Synthesis of Azu-Py-DF

To a solution of A3 (400 mg, 1.10 mmol) in dioxane/water (15/3 mL), B1 (1.3 g,2.51 mmol), K_2_CO_3_ (914 mg,6.61 mmol), and Pd(PPh_3_)_4_ (38 mg, 0.033 mmol) were added. The mixture was stirred at 110 °C under N_2_ for 16 h. The reaction was diluted with water (30 mL) and extracted with ethyl acetate (20 mL × 3). The organic phase was separated, washed with brine (20 mL × 2), dried over Na_2_SO_4_, concentrated under vacuum, and purified with column (10–25% ethyl acetate in petroleum ether) to afford Azu-Py-DF (450 mg, 41%) as a green solid. ^1^H NMR (400 MHz, DMSO-*d*_6_) δ 8.71 (d, *J* = 6.1 Hz, 2H), 8.57 (d, *J* = 10.3 Hz, 2H), 8.23 (s, 1H), 7.77 – 7.69 (m, 6H), 7.59 (d, *J* = 8.6 Hz, 4H), 7.50 (d, *J* = 7.4 Hz, 2H), 7.42 (d, *J* = 10.4 Hz, 2H), 7.34 – 7.22 (m, 6H), 7.17 (d, *J* = 9.0 Hz, 4H), 7.10 (d, *J* = 8.6 Hz, 4H), 7.04 – 6.95 (m, 6H), 3.77 (s, 6H), and 1.38 (s, 12H).

#### 2.1.4. Synthesis of Azu-Py-OMeTPA

To a solution of A3 (700 mg, 1.93 mmol) in dioxane/water(20/4 mL), TPA-Bpin (2.49 g,5.78 mmol), K_2_CO_3_ (1.60 g,11.57 mmol) and Pd(PPh_3_)_4_ (67 mg, 0.058 mmol) were added. The mixture was stirred at 110°C under N_2_ for 16 h. The reaction was diluted with water (50 mL) and extracted with ethyl acetate (30 mL × 3). The organic phase was separated, washed with brine (20 mL × 2), dried over Na_2_SO_4_, concentrated under vacuum, and purified with column (10–25% ethyl acetate in petroleum ether) to afford Azu-Py-OMeTPA (850 mg, 54%) as a green solid. ^1^H NMR (400 MHz, DMSO-*d*_6_) δ 8.71 (d, *J* = 6.1 Hz, 2H), 8.51 (d, *J* = 10.3 Hz, 2H), 8.15 (s, 1H), 7.71 (d, *J* = 6.1 Hz, 2H), 7.51 (d, *J* = 8.7 Hz, 4H), 7.38 (d, *J* = 10.4 Hz, 2H), 7.10 (d, *J* = 9.0 Hz, 8H), 6.98 – 6.91 (m, 12H), and 3.76 (s, 12H).

### 2.2. PSC Device Fabrication

In the fabrication of n-i-p-type PSC devices, a spin-coating technique was employed. Initially, laser-etched conductive fluorine-doped tin oxide (FTO) glass was cut into a 90 mm × 30 mm substrate and cleaned sequentially with deionized water, ethanol, and acetone for 15 min each. Next, a solution of 0.2 M titanium tetraisopropoxide and 2 M acetylacetone in isopropanol was applied onto the FTO substrate using an established spray pyrolysis method, followed by heating at 500 °C to form a compact oxide layer, which was then trimmed to 15 × 15 nm. After treating the surface with an ultraviolet ozone machine for 30 min, 30 μL of TiO_2_ solution dissolved in ethanol (mass ratio 6:1) was spin-coated onto the dense layer at 5000 rpm for 30 s. Mesoporous TiO_2_ was formed by annealing at 100 °C for 30 min, followed by a second annealing step at 500 °C. The perovskite precursor solution was prepared in a glove box by dissolving PbI_2_, FAI, and MACl in a 1.1:1:0.4 molar ratio in a mixture of N,N-dimethylformamide and dimethyl sulfoxide (volume ratio 4:1), stirred overnight at room temperature, and reserved for the subsequent spin-coating process. Then, 30 μL of the perovskite solution was spun onto the mesoporous TiO2 at 1000 rpm for 10 s, increased to 5000 rpm for 30 s, with 200 μL of chlorobenzene anti-solvent dripped onto the perovskite layer during the final 15 s. The resulting perovskite film was transferred to a hotplate at 150 °C and annealed for 30 min. An HTM solution consisting of 40 mg Azu-Py-OMeTPA/Azu-Py-DF, 30 mM LiTFSI, and 200 mM 4-tert-butylpyridine dissolved in 1 mL chlorobenzene was spin-coated onto the perovskite film surface at 4000 rpm for 30 s. Finally, a 100 nm thick gold layer was deposited onto the device film through vacuum evaporation.

## 3. Results and Discussion

### 3.1. Design and Synthesis

**Figure 1:**
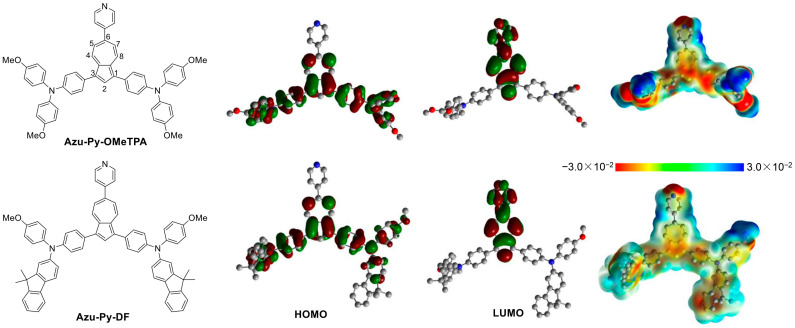
DFT and ESP images of Azu-Py-DF and Azu-Py-OMeTPA.

**Scheme 1:**
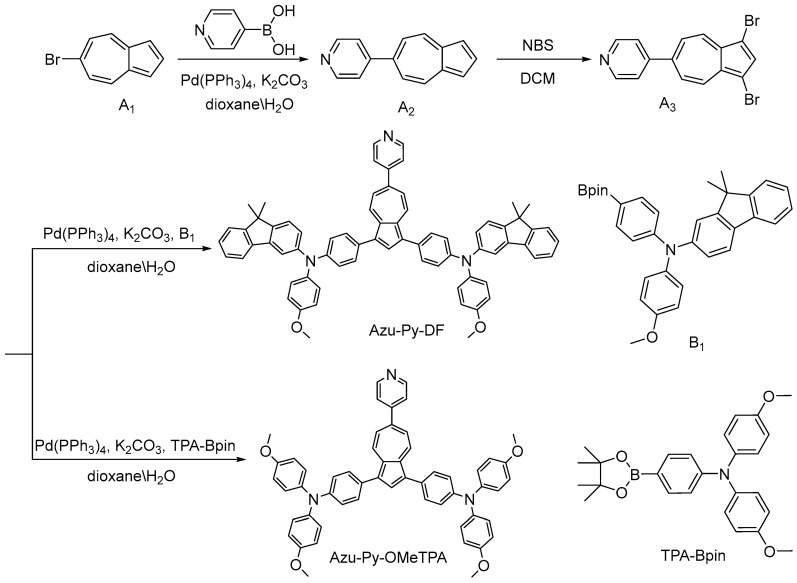
Synthetic routes of Azu-Py-DF and Azu-Py-OMeTPA.

The chemical structures of Azu-Py-DF and Azu-Py-OMeTPA are shown in Figure 1, and their synthetic routes are depicted in Scheme 1. The 4-pyridyl group at the 6-position was first introduced via Suzuki–Miyaura coupling with moderate yields from commercially available 6-bromoazulene. Subsequently, electron-donating triphenylamine derivatives at the 1- and 3-positons were synthesized through bromination followed by Suzuki–Miyaura coupling, achieving good yields. Detailed synthetic procedures and characterization data for the intermediates and final products are provided in the Experimental Section, while the ^1^H NMR spectra are included in the Supporting Information. The two electron-donating peripheral triphenylamine derivatives were synthesized according to previously reported procedures [29,30].

### 3.2. Photophysical and Electrochemical Properties

Density functional theory (DFT) and electrostatic potential (ESP) calculations were performed at the B3LYP/6-31G* level to determine the spatial distribution of HOMO and LUMO, as well as the structures and ESP images of Azu-Py-DF and Azu-Py-OMeTPA. As depicted in Figure 1, the introduction of the 4-pyridyl moiety at the 6-position of azulene is expected to improve the solubility and the film morphology of the material due to the different spatial orientations of the 4-pyridyl group and the azulene core. Analysis of molecular frontier orbitals revealed that the HOMO energy levels of Azu-Py-OMeTPA and Azu-Py-DF are mainly localized on the 1,3,5,7-positons of azulene core and the peripheral groups at the 1,3-position, while the LUMOs of both compounds are distributed on the 2,4,6,8-positions of the azulene core and the pyridine unit at the 6-position. This is expected because the 1,3-positions of azulene have large HOMO coefficients, while the 6-positon has large LUMO coefficients. ESP analysis indicated that the negative charge in both Azu-Py-DF and Azu-Py-OMeTPA is concentrated on the nitrogen atom of the pyridine group, which may interact and coordinate with unbonded Pd^2+^ defects at the perovskite interface, potentially enhancing the stability of the PSC devices [31].

**Figure 2:**
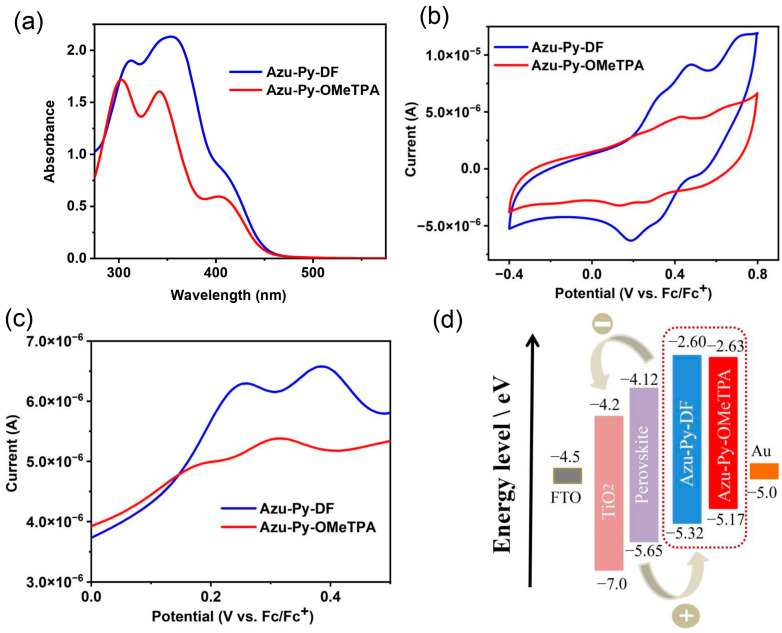
(**a**) UV–vis absorption, (**b**) CV curves, (**c**) normalized differential pulse voltammetry spectra, and (**d**) schematic diagram of the energy level arrangement of PSCs of Azu-Py-DF and Azu-Py-OMeTPA.

As shown in Figure 2a, the UV–vis absorption spectra of Azu-Py-DF and Azu-Py-OMeTPA in tetrahydrofuran (2 × 10^−5^ mol/L) solution were measured to elucidate their optical characteristics and band gaps. The maximum absorption peaks, centered at 413 nm and 404 nm, respectively, are associated with the intramolecular charge transfer from the peripheral donor to the acceptor core. The prominent peaks around 350–360 nm arise from π-π* electron transition of the triphenylamine-derived peripheral donor groups. Both compounds exhibit weak-to-negligible absorption in the visible light region, resulting in minimal competitive absorption with perovskite and HTMs. According to UV–vis spectra, the optical band gap (*E_g_*) of Azu-Py-DF and Azu-Py-OMeTPA were calculated as 2.72 eV and 2.54 eV, respectively, using the equation *E_g_* = 1240/λ_onset_. Both Azu-Py-DF and Azu-Py-OMeTPA exhibit red-shifted absorption compared to the previously reported 1,3-disubsitutited azulene derivatives [30], confirming that the introduction of the 4-pyridyl group effectively extended molecular conjugation. The HOMO energy levels were determined by cyclic voltammetry (CV) measurements, conducted in tetrahydrofuran solution with [TBA]PF_6_ (0.1 M) as electrolyte and ferrocene/ferrocenium (*Fc/Fc^+^*) as an external reference. The HOMO levels were calculated using the equation HOMO = −4.44 − [0.67 + (*E*^1/2^*_o x_ *− *E*^1/2^*_Fc_*_/*Fc*+_)], where *E*^1/2^*_ox_* and *E*^1/2^*_Fc_*_/*Fc*+_ represent half of the first redox potential of HTMs and ferrocene, respectively. As shown in Figure 2b, the HOMO levels for Azu-Py-DF and Azu-Py-OMeTPA are −5.32 eV and −5.17 eV, respectively. By utilizing the formula *E_LUMO_* = *E_HOMO_* + *E_g_*, the LUMO energy levels for Azu-Py-DF and Azu-Py-OMeTPA were calculated as −2.60 eV and −2.63 eV, respectively. The HOMO energy level of Azu-Py-OMeTPA is attributed to the presence of additional 4-methoxyphenyl groups on the triphenylamine peripheral groups, which induce a p-π conjugation effect. This enhances electron density on the phenyl ring, making Azu-Py-OMeTPA more susceptible to oxidation. The computational energy levels agreed with the differential pulse voltammetry (DPV) calculation results. The energy level diagram (Figure 2d) illustrates that the HOMO energy levels of Azu-Py-DF and Azu-Py-OMeTPA are higher than the valence band (−5.65 eV) of perovskite, providing sufficient driving force for hole extraction from the perovskite. Moreover, there is a significant barrier between the LUMO levels of these HTMs and the conduction band (−4.12 eV) of perovskite. Thus, both molecules effectively block the transfer of photogenerated electrons from perovskite to the Au electrode, fulfilling the basic requirements for HTMs in PSCs.

### 3.3. Photovoltaic Properties

**Figure 3:**
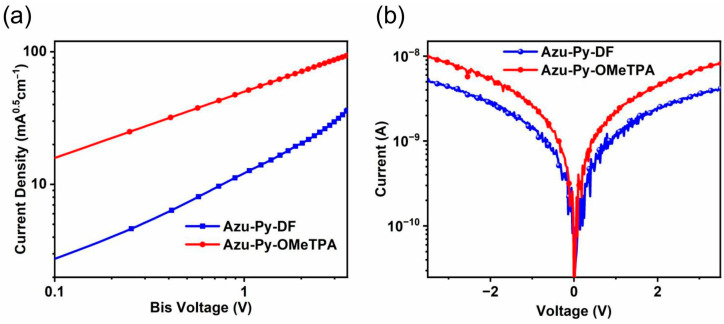
(**a**) Hole mobility measurement and (**b**) conductivity measurement of HTM Azu-Py-DF and Azu-Py-OMeTPA.

To further investigate the differences in charge transport properties of these HTMs, the hole mobilities of Azu-Py-DF and Azu-Py-OMeTPA were evaluated using the space-charge-limited current (SCLC) method; the adopted device structure is FTO/PEDOT:PSS/HTM/Au. Two devices, each with eight groups of data, were tested, and the final values represent the averaged data. The SCLC was determined using the following equation: *J* = (9/8)*με_o_*ε_r_(*V*^2^/*D*^3^), where *μ* is the hole mobility, *ε_o_* is the vacuum permittivity (8.85 × 10^−12^ F/m), *ε_r_* is the dielectric constant of the material (normally taken to approach 3 for organic semiconductors), *V* is the applied bias, *D* is the film thickness, and *J* stands for current density. As shown in Figure 3a, the hole mobilities were determined to be 3.16 × 10^−5^ cm^2^ V^−1^ s^−1^ and 1.47 × 10^−4^ cm^2^ V^−1^ s^−1^ for Azu-Py-DF and Azu-Py-OMeTPA, respectively, under the same conditions. It was observed that the hole mobility of Azu-Py-OMeTPA was significantly higher than that of Azu-Py-DF. For conductivity measurements, two devices with six sets of data were collected, and the final values were based on the averaged data. Two-probe electrical conductivity measurements were conducted to assess the electrical conductivities of the HTM films (Figure S2). The electrical conductivity (σ) was calculated by using the following equation: *σ* = *W*/(*RLD*), where *L* stands for the channel length (10 mm), *W* stands for the channel width (2 mm), *D* is the combined film thickness of the HTM and TiO_2_, and *R* is the film resistance value derived from the gradients of the curves. The conductivity of Azu-Py-OMeTPA was determined to be 5.01 × 10^−5^ S cm^−1^, higher than that of Azu-Py-DF (2.60 × 10^−5^ S cm^−1^). This improvement could be attributed to the optimization of the spatial configuration of Azu-Py-OMeTPA molecules upon the introduction of triphenylamine peripheral groups, which enhances solubility and improves thin film morphology. Nevertheless, both HTMs still exhibit lower mobility and conductivity than Spiro-OMeTAD (Table 1) [30], possibly due to persistent flaws in their thin film, as discussed below.

**Figure 4:**
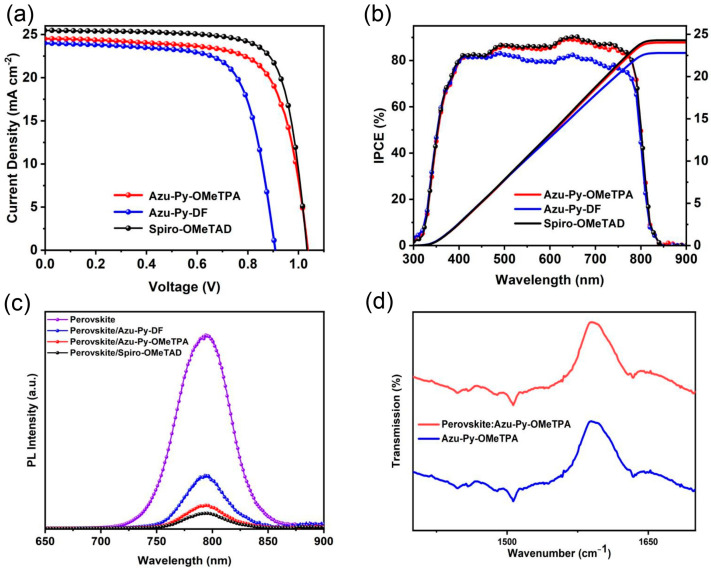
(**a**) *J*–*V* curves and (**b**) IPCE spectra of PSC devices based on azulene-based HTM, (**c**) steady-state PL spectra of perovskite and perovskite/HTM films, and (**d**) infrared spectrum of Azu-Py-OMeTPA and the mixed sample of Azu-Py-OMeTPA/perovskite.

The *J-V* (current density–voltage) curves of the perovskite solar cells (PSCs) employing the synthesized compounds Azu-Py-DF and Azu-Py-OMeTPA as HTMs are displayed in Figure 4a. The incident photon-to-current conversion efficiency (IPCE) graphs of PSCs are presented in Figure 4b, with detailed parameters summarized in Table 2. Under AM 1.5 G illumination, the PCE of the PSC based on Azu-Py-OMeTPA reached 18.10% (*J_sc_* = 24.48 mA cm^−2,^ *V_oc_* = 1.04 V, *FF* = 71.42%), while the PSC with Azu-Py-DF exhibited a slightly lower PCE of 15.39% (*J_sc_* = 23.97 mA cm^−2^, *V_oc_* = 0.91 V, *FF* = 70.68%). Under the same conditions, the Spiro-OMeTAD-based PSC achieved a PCE of 20.00% (*J_sc_* = 25.47 mA cm^−2,^ *V_oc_* = 1.03 V, *FF* = 76.06%) [30]. Although Azu-Py-OMeTPA outperforms Azu-Py-DF, it remains less competitive than Spiro-OMeTAD due to suboptimal film morphology. The calculated short-circuit current densities (*J_sc_*^cal^) for devices based on Azu-Py-DF, Azu-Py-OMeTPA, and Spiro-OMeTAD were 24.03 mA cm^−2^, 22.79 mA cm^−2^, and 24.28 mA cm^−2^, respectively, which are consistent with the parameters obtained from the *J-V* curves. Azu-Py-OMeTPA shows significantly higher efficiency than Azu-Py-DF from 480 nm to 800 nm, attributable to its superior hole mobility and film-formation property, which reduce charge recombination loss at the interface. In contrast, the thin film of Azu-Py-DF contains more defects, leading to more severe recombination, especially in the visible wavelength region. For reference, the highest performance for 1,3-disubsituted counterpart is 15.41% [30].

Steady-state photoluminescence (PL) spectra of pristine perovskite and perovskite coated with HTMs films were measured to evaluate differences in charge transport properties. As shown in Figure 4c, coating the perovskite with Azu-Py-OMeTPA resulted in significant quenching of the fluorescence intensity. Spiro-OMeTAD exhibited the highest quenching efficiency. The degree of fluorescence quenching is attributed to thin film morphology and carrier transport capability, which effectively promotes hole extraction and transport. It is noteworthy that almost no shift in the emission peaks was observed in the PL spectra, indicating that the 4-pyridyl group did not passivate perovskite through chemical interaction, contrary to initial assumptions. Figure 4d presents the infrared spectra of pure Azu-Py-OMeTPA and a mixed sample of Azu-Py-OMeTPA with perovskite, showing no noticeable peak shifts, further confirming the absence of chemical interaction between the 4-pyridyl group and perovskite. This may be because the 4-pyridyl group is substituted at the electron-deficient seven-membered ring of azulene, reducing its electron donating ability and preventing coordination with Pb^2+^.

### 3.4. Surface Morphologies and Hydrophobicities

**Figure 5:**
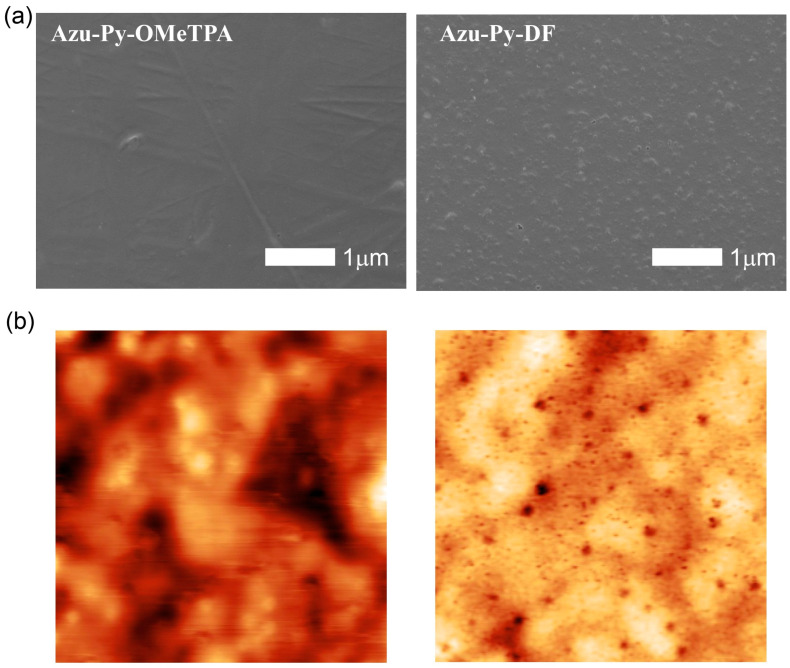
(**a**) SEM and (**b**) AFM of HTMs spin-coated on perovskite layer.

Scanning electron microscopy (SEM) and atomic force microscopy (AFM) were utilized to initially assess the surface characteristics of films made from Azu-Py-DF and Azu-Py-OMeTPA coated on perovskite, which play a crucial role in determining the performance of PSC. As shown in Figure 5, SEM images reveal that the thin film of Azu-Py-DF contains numerous pinholes, probably due to its insufficient solubility and weaker intermolecular interactions, which may result in severe charge recombination, ultimately limiting PSC efficiency. In comparison, although not perfect, the thin film quality of Azu-Py-OMeTPA is significantly improved, with fewer defects. This improvement may be attributed to the presence of additional methoxy groups in the triphenylamine peripheral units, which enhance intermolecular interactions, solubility, and film formation property. The enhanced film-forming capability of Azu-Py-OMeTPA was further validated by AFM imaging, aligning with its superior device performance. Moreover, the film morphology of Azu-Py-OMeTPA is noticeably superior to that of its previously reported 1,3-disubsituted counterpart [30].

**Figure 6:**
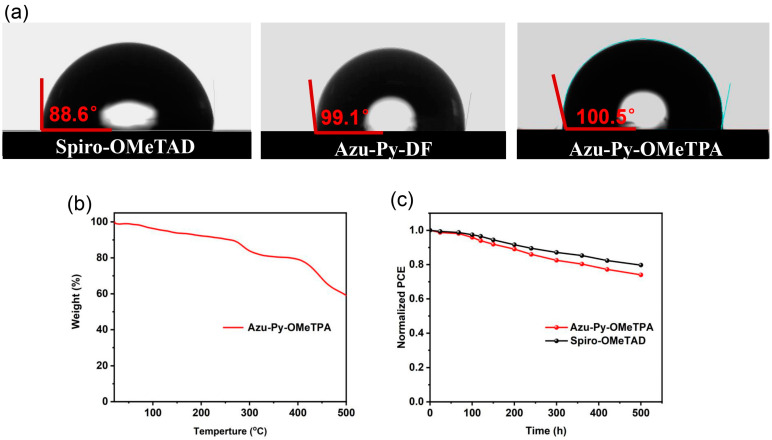
(**a**) Water contact angle test for films of different materials, (**b**) TGA, and (**c**) stability of Azu-Py-OMeTPA-based PSC.

Contact angle measurements of Azu-Py-DF, Azu-Py-OMeTPA, and Spiro-OMeTAD, spin-coated onto perovskite thin films, are shown in Figure 6 to evaluate their hydrophobicity. The water contact angles were measured as 99.1° for Azu-Py-DF and 100.5° for Azu-Py-OMeTPA, both slightly higher than that of Spiro-OMeTAD (88.6°). This improved hydrophobicity may help mitigate perovskite degradation under certain humid conditions. However, thermogravimetric analysis (TGA) revealed that Azu-Py-OMeTPA exhibits a mass loss of less than 5% around 300 °C, indicating a lower thermal stability compared to Spiro-OMeTAD [32], suggesting greater sensitivity to heat. The long-term stability of the unpackaged devices under ambient conditions (RH 40–50%, room temperature) is presented in Figure 6c. The PSCs based on Azu-Py-OMeTPA maintained 74% of their initial efficiency after 500 h of aging, which is lower than that of Spiro-OMeTAD (80%) under the same conditions. This indicates that further studies are necessary to improve the stability of azulene-based HTMs.

## 4. Conclusions

In summary, two novel compounds based on 6-(4-pyridyl)azulene, Azu-Py-DF and Azu-Py-OMeTPA, were synthesized and applied as HTMs in PSC. The introduction of the 4-pyridyl group at the 6-position of azulene improved solubility, reduced crystallinity and adjusted energy levels. Both compounds exhibit suitable HOMO and LUMO levels, good hole mobility, conductivity, and hydrophobicity. The thin film quality of Azu-Py-OMeTPA was superior to that of Azu-Py-DF due to the additional methoxy groups, which strengthened intermolecular interaction and promoted mobility, although there is still room for improvement. PSCs based on Azu-Py-OMeTPA achieved a PCE of 18.10% with *V_oc_* comparable to that of Spiro-OMeTAD. The lower *J_sc_* and *FF* were attributed to minor thin film defects, which could be mitigated through further chemical modification. However, the initial anticipated passivation effect of the 4-pyridyl group was hampered by the electron-deficient seven-membered ring of azulene. This work highlights the potential of azulene derivatives as HTMs due to their unique structural characteristics and favorable charge transport properties. Substituting the azulene core with electron-rich substituents that offer stronger passivation effects could be a promising strategy. We hope that this study serves as a reference for the development of azulene-based functional materials.

## Figures and Tables

**Table 1:** Optical and electrochemical parameters of Azu-Py-DF and Azu-Py-OMeTPA.

HTM	*λ_abs_* (nm)	*E_g_* (eV)	HOMO (eV)	LUMO (eV)	Hole Mobility (cm^2^ V^−1^ S^−1^)	Conductivity (S cm^−1^)
Azu-Py-DF	413	2.72	−5.32	−2.60	3.16 × 10^−5^	2.60 × 10^−5^
Azu-Py-OMeTPA	404	2.54	−5.17	−2.63	1.47 × 10^−4^	5.01 × 10^−5^
Spiro-OMeTAD [30]	386	2.99	−5.09	−2.10	1.86 × 10^−4^	7.38 × 10^−5^

**Table 2:** Photovoltaic performances of PSCs based on Azu-Py-DF and Azu-Py-OMeTPA.

HTM	*V_oc_* (V)	*J_sc_* (mA cm^−2^)	*FF* (%)	PCE (%)	*J_sc_^cal^* (mA cm^−2^)
Azu-Py-OMeTPA	1.04	24.48	71.42	18.10	24.03
Azu-Py-DF	0.91	23.97	70.68	15.39	22.79
Spiro-OMeTAD [30]	1.03	25.47	76.06	20.00	24.28

## Data Availability

The original contributions presented in this study are included in the article/Supplementary Material. Further inquiries can be directed to the corresponding authors.

## Supplementary Materials

The following supporting information can be downloaded at: https://www.mdpi.com/article/10.3390/ma18071400/s1. The HOMO/LUOMO distribution of azulene, schematic illustrations of conductivity device, ^1^H NMR spectra of compounds, flowchart of device fabrication, photo of perovskite solar cell, and nomenclature table are shown in Supporting Information. Figure S1. The chemical structure, resonance structure and HOMO/LUMO distribution of azulene. Figure S2. Schematic illustrations of the conductivity device: (a) top-sectional view; (b) cross-sectional view. Figure S3. ^1^H NMR of A2 in CDCl_3_. Figure S4. ^1^H NMR of A3 in CDCl_3_. Figure S5. ^1^H NMR of Azu-Py-DF in DMSO-d6. Figure S6. ^1^H NMR of Azu-Py-OMeTPA in DMSO-d6. Figure S7. The flowchart of device fabrication and the photo of a perovskite solar cell (top view and bottom view).
