# Supplementary material for: 6-(4-Pyridyl)Azulene Derivatives as Hole Transport Materials for Perovskite Solar Cells
Source: Materials (Basel). 2025 Mar 21;18(7):1400. doi: 10.3390/ma18071400 (PMC11989244; doi:10.3390/ma18071400)
Supplement: Supplementary file 1 [file materials-18-01400-s001.zip › materials-3468218-supplementary.pdf]

Supporting information

6-(4-Pyridyl)Azulene Derivatives as Hole Transport Materials for Perovskite Solar Cells

Yuanqing Sun [1], Zhangyan Wang [2], Tianyi Geng [3], Xinyue Liu[1], Yangyang Su [1], Yi Tian [1,*], Ming Cheng [1,] Hongping Li [2,*],

[1] Institute for Energy Research, Jiangsu University, Zhenjiang 212013, China.

[2] Institute for Advanced Materials, School of Materials Science and Engineering, Jiangsu University, Zhenjiang, 212013, China.

[3] School of Energy and Power Engineering, Jiangsu University, Zhenjiang 212013, China.

** E-mail:* tianyi@ujs.edu.cn *(Y. Tian);*

** E-mail:* hpli@ujs.edu.cn *(H. Li);*

Analysis instruments and conditions

The ^{1}H NMR spectra of HTMs were recorded using a Bruker 400 MHz instruments. DFT calculations were performed by using the Gaussian program at the B3LYP/6-31G* level. The UV–Vis absorption spectra were measured with a Shimadzu UV-2450 spectrophotometer. CV was performed in tetrahydrofuran with $[TBA]PF_6$ as the supporting electrolyte, an $Ag^+/AgNO_3$ electrode as the reference electrode, a carbon-glass electrode as the working electrode, a Pt electrode as the counter electrode, and ferrocene/ferrocenium (Fc/Fc^+) as an internal reference, using a CH Instruments electrochemical workstation (model 660 A). SEM images were obtained using a JEOL JSM-7800 microscope. The light source for the *J-V* measurement was an AM 1.5G solar simulator, with an incident light intensity of 100 mW/cm^2 calibrated with a standard Si solar cell. The tested solar cells were masked to a working area of 0.09 cm^2. IPCE was performed with Newport QEPVSI-B photoelectric chemical quantum efficiency testing and analysis system.

Chemical and materials

Li-bis(trifluoromethanesulfonyl) imide (Li-TFSI) and 4-tert-butylpyridine (tBP) were purchased from Xi'an Polymer Light Technology Corp.. Spiro-OMeTAD, TiO_2 paste (30NRD) and fluorine doped tin oxide (FTO) coated glass were purchased from Advanced Election Technology Co., 1,4-dioxane, potassium carbonate (K_2CO_3), and chlorobenzene (CB) were purchased from SigmaAldrich. Palladium catalysts were purchased from Shanghai Haohong Scientific Co., Ltd. Other raw materials used for synthesis such as pyridine-4-boronic acid, 4-(4,4,5,5-tetramethyl-1,3,2-dioxaborolan-2-yl)-N,N-bis(4-methoxyphenyl)aniline, N-Bromosuccinimide and 2-Bromo-9,9′-dimethylfluorene were purchased from Energy Chemicals. All these materials in experiments were used as received without any further purification.

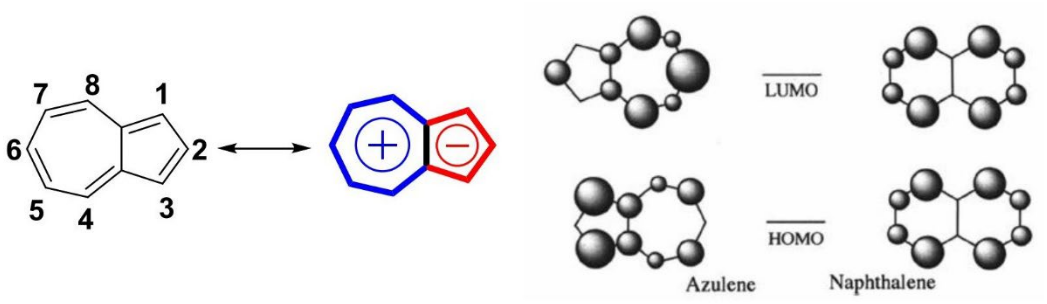

Figure S1. The chemical structure, resonance structure and HOMO/LUMO distribution of azulene.

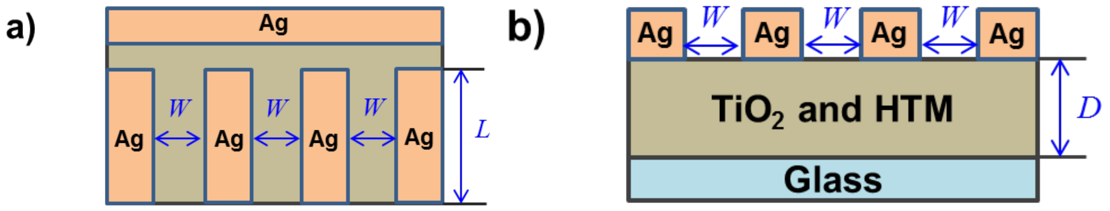

Figure S2. Schematic illustrations of the conductivity device: (a) top-sectional view; (b) cross-sectional view

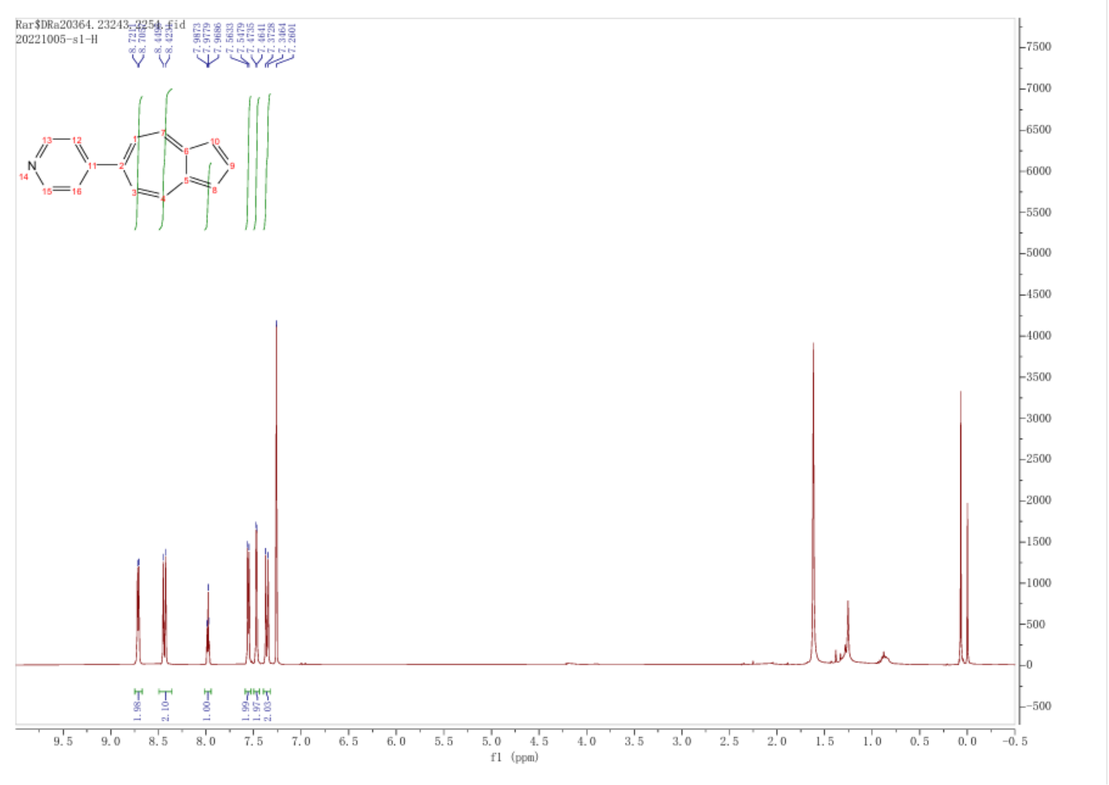

Figure S3 ^{1}H NMR of A2 in $CDCl_3$

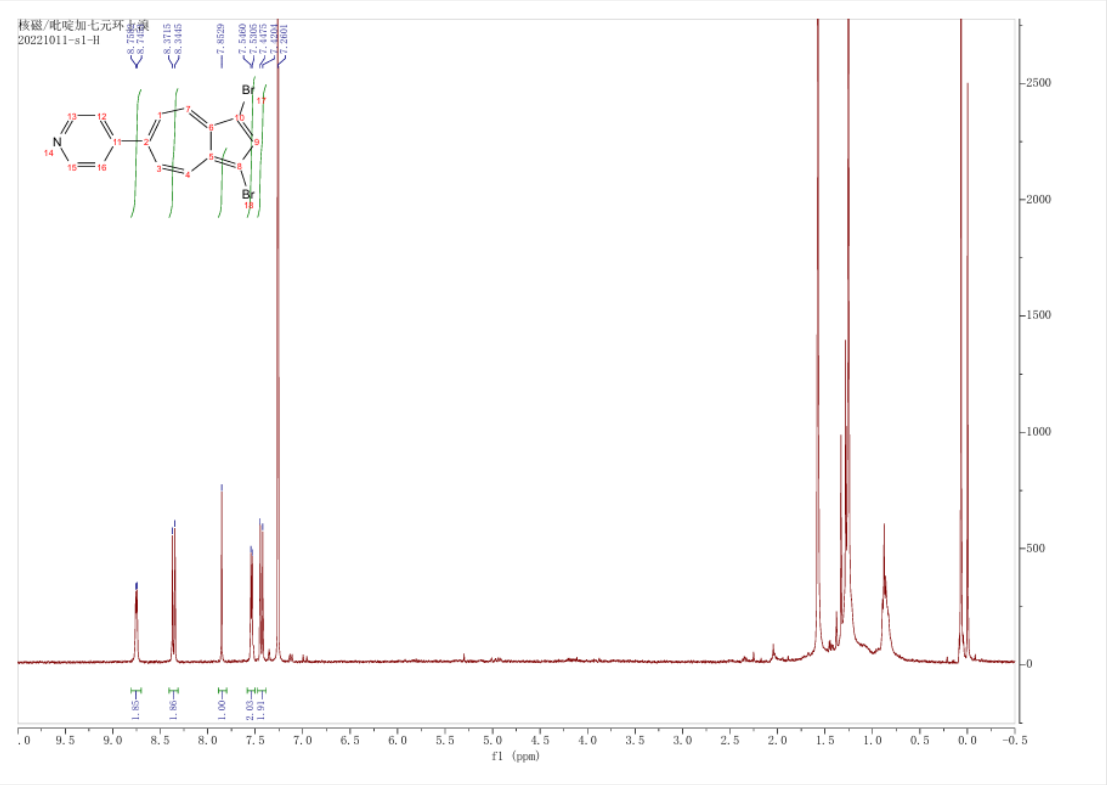

Figure S4 ^{1}H NMR of A3 in $CDCl_3$

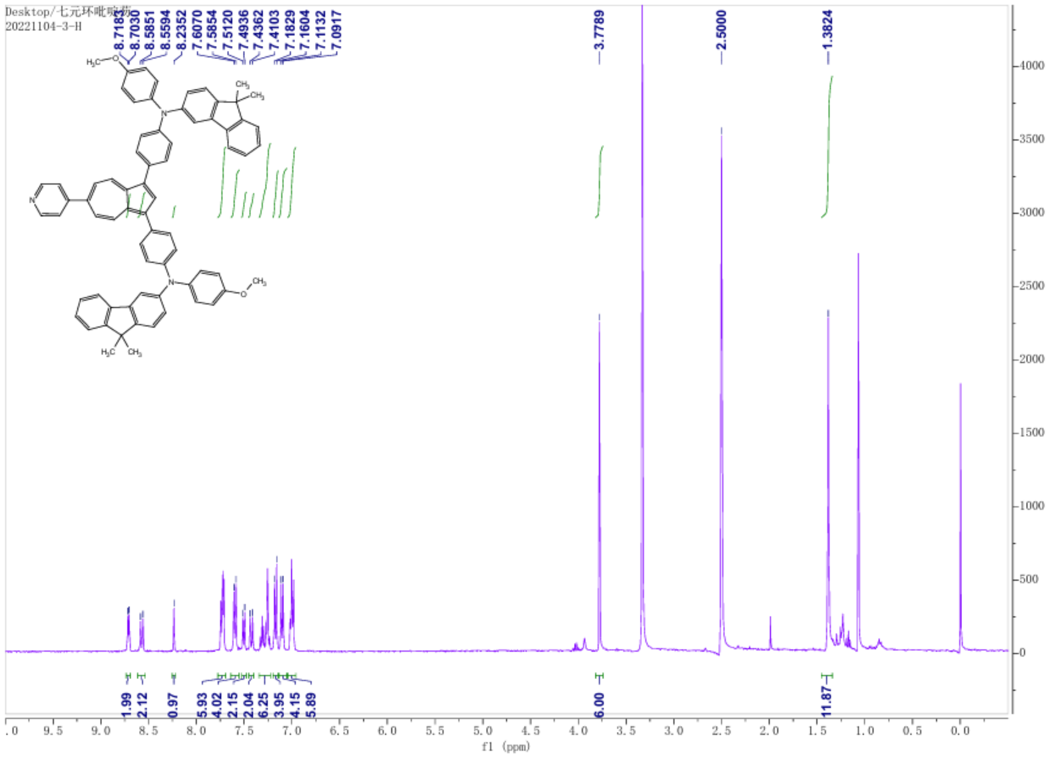

Figure S5 ¹H NMR of Azu-Py-DF in DMSO-d6

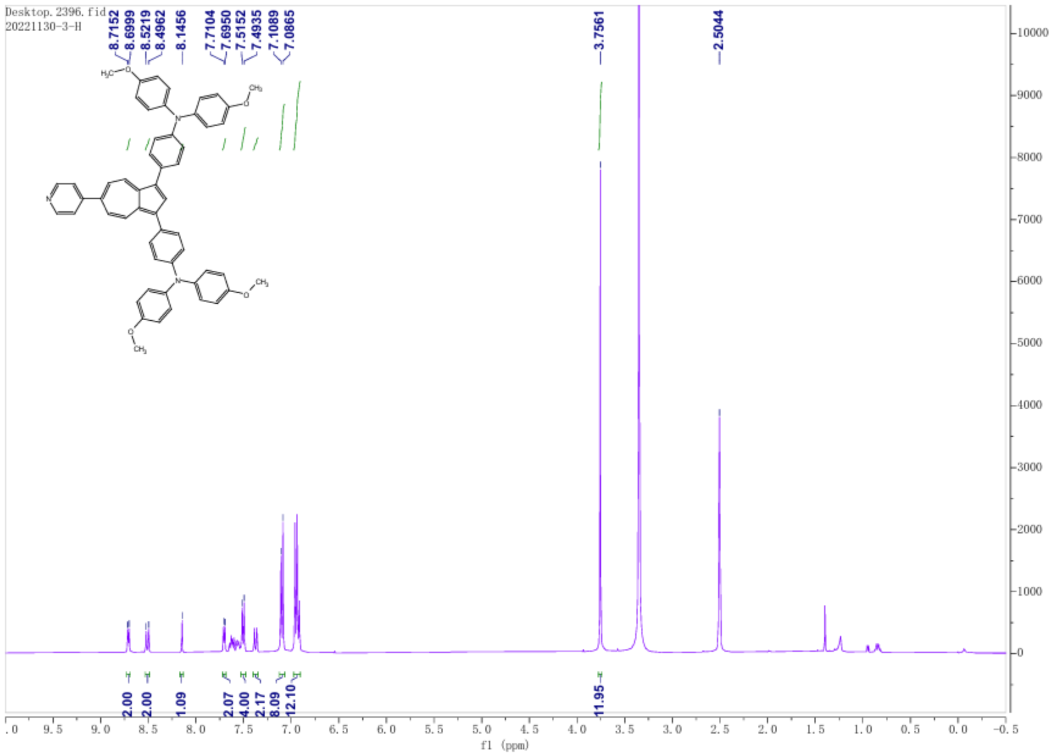

Figure S6 ¹H NMR of Azu-Py-OMeTPA in DMSO-d6

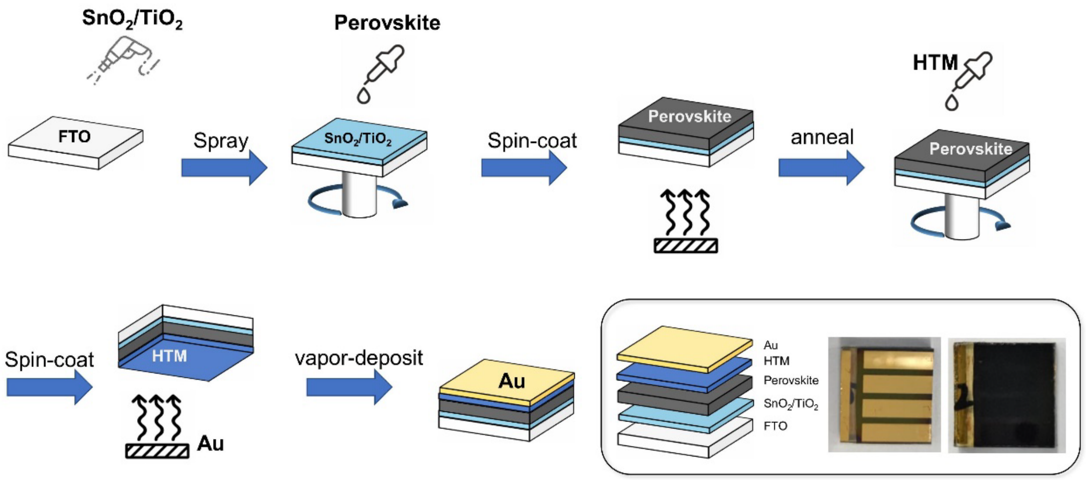

Figure S7 The flowchart of device fabrication and the photo of a perovskite solar cell (top view and bottom view).

Nomenclature Table

Azu-Py-DF	name of target molecule, its structure was described in scheme 1
Azu-Py-OMeTPA	name of target molecule, its structure was described in scheme 1
A_1, A_2, A_3, B_1, B_2	code name for intermediate product and certain chemical structure, detailed structures were shown in scheme 1
TPA-Bpin	4-(4,4,5,5-Tetramethyl-1,3,2-dioxaborolan-2-yl)-N,N-bis(4-methoxyphenyl)aniline, its structure was described in scheme 1
PSC	perovskite solar cell
HTM	hole transport material
HOMO	highest occupied molecular orbital
LUMO	lowest unoccupied molecular orbital

Spiro-OMeTAD	*2,2',7,7'*-tetrakis(N,N-*p*-dimethoxyphenylamino)-*9,9'*-spirobifluorene
FTO	fluorine doped tin oxide
TBP	4-tert-butylpydrdine
DFT	Density functional theory
ESP	electrostatic potential
E_g	optical band gap
CV	cyclic voltammetry
DPV	differential pulse voltammetry
UV–vis	ultraviolet–visible spectroscopy
PEDOT:PSS	poly(3,4-ethylenedioxythiophene) polystyrene sulfonate
V_{oc}	open circuit voltage
J_{sc}	short-circuit current
FF	fill factor
PCE	power conversion efficiency
SCLC	space-charge-limited current
